# Dynamic changes in the systemic immune-inflammation index predict the prognosis of EGFR-mutant lung adenocarcinoma patients receiving brain metastasis radiotherapy

**DOI:** 10.1186/s12890-022-01866-7

**Published:** 2022-03-03

**Authors:** Qi Wang, Xiaojing Tan, Guangchuan Deng, Shuai Fu, Jianbin Li, Zhenxiang Li

**Affiliations:** 1 Department of Radiation Oncology, Shandong Cancer Hospital and Institute, Shandong First Medical University and Shandong Academy of Medical Sciences, Jinan, 250117 China; 2 Shandong First Medical University and Shandong Academy of Medical Sciences, Jinan, China; 3 Department of Oncology, Dongying People’s Hospital, Dongying, China; 4 Department of Medical Oncology, Shandong Cancer Hospital and Institute, Shandong First Medical University and Shandong Academy of Medical Sciences, Jinan, China

**Keywords:** Systemic immune-inflammation index, Dynamic change, Epidermal growth factor mutation, Brain metastases, Brain radiotherapy

## Abstract

**Background:**

The systemic immune-inflammation index (SII) has recently emerged as a predictor of survival in non-small cell lung cancer patients. There is also tight correlation between radiotherapy and immune status, and brain metastases (BM) radiotherapy is an important treatment in patients with BM from lung adenocarcinoma harboring epidermal growth factor receptor (EGFR) mutations. Hence, this study aimed to present the prognostic value of SII and its dynamic changes during BM radiotherapy in EGFR-mutant lung adenocarcinoma patients with BM.

**Methods:**

Patients with EGFR-mutant lung adenocarcinoma who received BM radiotherapy between November 2011 and April 2021 were included in this retrospective study. The SII was calculated using data acquired within 1 week before the start of radiation treatment and 1 week before its completion. According to the cutoff value of SII before radiation treatment determined using receiver operating characteristic curve analyses, we divided the patients into a high group and a low group. Patients were further classified into high–high, high–low, low–low, and low–high groups based on dynamic changes in SII. Prognostic values of the SII and other factors were determined using the Kaplan–Meier method, as well as univariate and multivariate Cox analysis.

**Results:**

A total of 202 patients met the inclusion criteria, and the median overall survival (OS) of the entire cohort was 36 months. According to the SII cutoff of 859.79, an SII value below this cutoff was associated with longer OS (hazard ratio 0.6653, 95% confidence interval 0.4708–0.9402, *P* < 0.05). The patients in the low–low group, whose SII within 1 week before the start and end of BM radiotherapy were below the cutoff, had a median OS of 55.2 months, which was significantly longer than the OS in all other groups (*P* < 0.05). Univariate and multivariate analyses confirmed that dynamic SII change (*P* = 0.032), Lung-molGPA (*P* < 0.001), and thoracic radiation (*P* = 0.048) were independently correlated with OS.

**Conclusions:**

The SII and its dynamic change may have a prognostic value in patients with EGFR-mutant lung adenocarcinoma treated with BM radiotherapy.

## Background

The treatment of advanced lung adenocarcinoma has entered the era of molecular targeted therapy, and mutant epidermal growth factor receptors (EGFRs) are the most common therapeutic target. EGFR tyrosine kinase inhibitors (TKIs), as the first-line treatment for patients with EGFR-mutant lung adenocarcinoma, can significantly improve the prognosis and quality of life in this population [1]. Brain metastases (BM) occur in more than 40% of patients with non-small cell lung cancer (NSCLC), and in patients with advanced EGFR-mutant NSCLC, this incidence exceeds 60% in long-term survivors [2, 3]. Clinical data show that BM is associated with increased morbidity and mortality leading to a very poor prognosis. Brain radiotherapy is considered the cornerstone of BM treatment and remains a vital treatment for patients with brain metastases [4]. The median intracranial progression-free survival of patients with combination treatment (brain radiotherapy and EGFR-TKIs) was longer than that of patients receiving EGFR-TKIs alone (21.5 vs. 15.0 months) [5]. This suggests that the addition of brain radiotherapy facilitates controlling intracranial metastatic lesions. Reliable predictive factors are needed for predicting the survival and prognosis of these patients after craniocerebral radiotherapy to identify patient subgroups benefiting more from this treatment. This can help to determine the optimal therapeutic strategy and, thus, improve survival and quality of life in this patient population.

The immune system plays significant pro- and anti-tumorigenic roles at all stages of tumorigenesis. At present, it is generally believed that inflammation has a great influence on the composition of the tumor microenvironment, which includes cancer cells, fibroblasts, vascular cells, and inflammatory immune cells, particularly affecting the plasticity of tumor cells [6]. Several inflammation- and immune-based prognostic indices, developed to predict patient survival, are associated with overall survival (OS), for instance, the neutrophil–lymphocyte ratio, platelet–lymphocyte ratio, and lymphocyte–monocyte ratio [7–9]. Recently, the systemic immune-inflammation index (SII), which is calculated based on the lymphocyte, neutrophil, and platelet counts, has been shown to be a good prognostic factor in various types of tumors [10]. Many studies have demonstrated that the SII is a convenient and readily available test that serves as an independent predictor of OS for patients with various malignancies, such as colorectal cancer [11], hepatocellular carcinoma [12], pancreatic cancer [13], and NSCLC [14].

The tight correlation between radiotherapy and immune status has been confirmed and the prognostic value of SII in patients with BMs from EGFR-mutant lung adenocarcinoma who underwent brain radiotherapy remains elusive. Therefore, this study aimed to investigate whether the SII value before brain radiotherapy and its dynamic changes during brain radiotherapy were predictive for OS in patients with BMs from EGFR-mutant lung adenocarcinoma.

## Methods

### Patient selection

We screened patients diagnosed with NSCLC at Shandong Cancer Hospital and Institute between November 2011 and April 2021. The inclusion criteria were as follows: (1) pathologically diagnosed as primary lung adenocarcinoma; (2) harboring an EGFR mutation; (3) presence of brain metastases diagnosed by computed tomography or magnetic resonance imaging at the initial diagnosis or in the course of the disease; (4) previously received BM radiotherapy, including whole-brain radiotherapy (WBRT), local radiotherapy, or WBRT + Boost; (5) no other primary malignancies; and (6) complete records of blood test results both within 1 week before the start of the BM radiation treatment and within 1 week before its completion. This retrospective study was approved by the Ethics Committee of Shandong Cancer Hospital and Institute and was conducted in accordance with the Declaration of Helsinki. All the patients were diagnosed and treated in Shandong Cancer Hospital and Institute, so we obtained the permissions to access the data from the Ethics Committee of Shandong Cancer Hospital and Institute. Considering the retrospective nature of the study, the informed consent was waived.

### Data collection and definition

The following patient characteristics were included: sex, age, smoking history, EGFR mutation status, Lung-molGPA class, brain radiation mode, thoracic radiation, chemotherapy, metastatic sites and full blood count for calculating the dynamic SII change. These data were acquired from the electronic medical record system of Shandong Cancer Hospital. OS was defined as the time from the date of diagnosis to the date of death due to any reason or the last date of follow-up.

In this study, thoracic radiotherapy meant the radiotherapy for the primary lung tumors and thoracic metastatic lymph nodes. The dose of thoracic radiotherapy ranged from 2 Gy per fraction to 5.5 Gy per fraction, and the total dosage ranged from 27 to 75 Gy. Most patients received palliative radiotherapy, and only a small number received curative radiotherapy. All patients included in study previously received BM radiotherapy. In patients who treated with whole brain radiotherapy, the total radiotherapy dose ranged from 25 to 50 Gy (median prescribed dose 40 Gy). The range of local radiotherapy was 20–62.5 Gy (median prescribed dose 50 Gy). Additionally, in the WBRT + Boost group, the range of whole brain radiotherapy was 30–54 Gy (mediate prescribed dose 40 Gy) and the additional radiation boost for local metastases was 6–24 Gy (median prescribed dose 15 Gy).

Lung-molGPA is a new prognostic model for patients with brain Metastasis of EGFR-mutated NSCLC, which based on the patient’s age, KPS, the number of extracranial and BM, and the status of gene mutations. A score of 0–4 indicated significant impact on OS in patients. The grouping standard referred to our team's previous study, which revealed that a statistical difference in median survival between the two groups of BM patients from EGFR-mutant lung adenocarcinoma with a score of 1–2 and a score of 2.5–4 [15].

The SII, NLR, PLR and LMR were calculated using the following formulas: SII = platelet counts × neutrophil counts/lymphocyte counts, NLR = neutrophil counts/lymphocyte counts, PLR = platelet counts/lymphocyte counts, LMR = lymphocyte counts/monocyte counts. Blood counts were obtained at two time points: one within a week before the start of BM radiation treatment and another within a week before the completion of the radiotherapy. The SII cutoff value was determined using the blood counts before the radiation based on receiver operating characteristic (ROC) curve analysis. Patients were subsequently stratified into high and low SII groups. The prognostic significance of dynamic SII changes was explored in more detail in this study. According to the dynamic changes between SII within 1 week before the start of the BM radiation treatment and within 1 week before its completion, the study population was subsequently further divided into four subgroups: high–high group, high–low group, low–low group, and low–high group. The high–high group comprised patients who persistently had SII values above the cutoff, whereas patients who transitioned from high to low SII values were included in the high–low group. The remaining groups were defined in the same manner.

### Statistical methods

We performed all statistical analyses using SPSS software (version 25.0). ROC curves were generated to analyze the areas under the ROC curve, and the Youden Index was used to identify the optimal SII cutoff value. The relationship between SII and clinicopathological factors was analyzed using the chi-square test. The Kaplan–Meier method and the log-rank test were used to perform survival analyses and compare survival differences. The prognostic values of variables for OS were assessed using Cox proportional hazards regression and are expressed as *P* values, hazard ratios, and 95% confidence intervals. Variables with statistical significance in the univariate Cox analysis were included in the multivariate Cox analysis. A two-sided *P* value of < 0.05 was considered statistically significant.

## Results

### Patient characteristics

Baseline patient characteristics are summarized in Table 1. Ultimately, 202 patients met the inclusion criteria in this retrospective study, including 73 (36.1%) men and 129 (63.9%) women. In this study population with a median age of 54 (28–81) years, 44 (21.8%) of the patients had a smoking history, whereas 158 (78.2%) had never smoked. The majority of patients had EGFR mutations in exons 19 (83, 41.09%) and 21 (93, 46.04%). Other rare mutations, including exon 18 and 20 mutations, accounted for a small proportion (12, 5.94%). 14 (6.93%) unclear ones meant that patients with identified EGFR sensitive mutations who responded well to EGFR-TKI treatment whereas it is not clear of the exon site of mutation due to negligence of medical record writer. According to the Lung-molGPA classification system, the numbers of patients with scores 0–2 and 2.5–4 were 89 (44.1%) and 113 (55.9%), respectively. All patients received brain metastases radiotherapy: 76 (37.6%) patients received WBRT, 89 (44.1%) local radiotherapy, and 37 (18.3%) WBRT + Boost. There were 79 patients who received thoracic radiation and 168 ones who received chemotherapy. In addition to brain metastasis in all patients, 96, 129, 37, 41, 52 and 30 patients developed intrapulmonary metastasis, bone metastasis, liver metastasis, adrenal metastasis, pleural metastasis and others respectively. Other metastases included kidney, pancreas, spleen, spinal cord, and soft tissue, which are aggregated together because of the small number.

**Table 1:** Baseline characteristics of all 202 patients stratified by SII before brain radiotherapy

Characteristics	Variables	N (%)	SII			
			< 859.79	≥ 859.79	X2	*P*
Sex	Female	129 (63.9)	72	57	0.494	0.482
	Male	73 (36.1)	37	36		
Age (years)	< 60	140 (69.3)	77	63	0.198	0.656
	≥ 60	62 (30.7)	32	30		
Smoking status	Never	158 (78.2)	87	71	0.355	0.551
	Former/current	44 (21.8)	22	22		
EGFR mutation	Exon 21	93 (46.04)	46	46	6.542	0.088
	Exon 19	83 (41.09)	50	33		
	Other (18 or 20)	12 (5.94)	3	9		
	Unclear	14 (6.93)	9	5		
Lung-molGPA	0–2	89 (44.1)	51	38	0.716	0.398
	2.5–4	113 (55.9)	58	55		
Brain radiation mode	WBRT	76 (37.6)	44	32	0.760	0.684
	Local radiotherapy	89 (44.1)	46	43		
	WBRT + Boost	37 (18.3)	19	18		
Thoracic radiation	Yes	79 (39.1)	43	36	0.012	0.914
	No	123 (60.9)	66	57		
Chemotherapy	Yes	169 (83.7)	93	76	0.476	0.490
	No	33 (16.3)	16	17		
Metastatic sites	Lung	96 (47.5)	50	46	1.183	0.947
	Bone	129 (63.9)	66	63		
	Liver	37 (18.3)	17	20		
	Adrenal gland	41 (20.3)	19	22		
	Pleura	52 (25.7)	27	25		
	Others	30 (14.9)	17	13		
SII	< 859.79	109 (54)	NA	NA	NA	NA
	≥ 859.79	93 (46)	NA	NA		
Dynamic change of SII	High–high	59 (29.2)	NA	NA	NA	NA
	High–low	34 (16.8)	NA	NA		
	Low–low	67 (33.2)	NA	NA		
	Low–high	42 (20.8)	NA	NA		

**Fig. 1:**
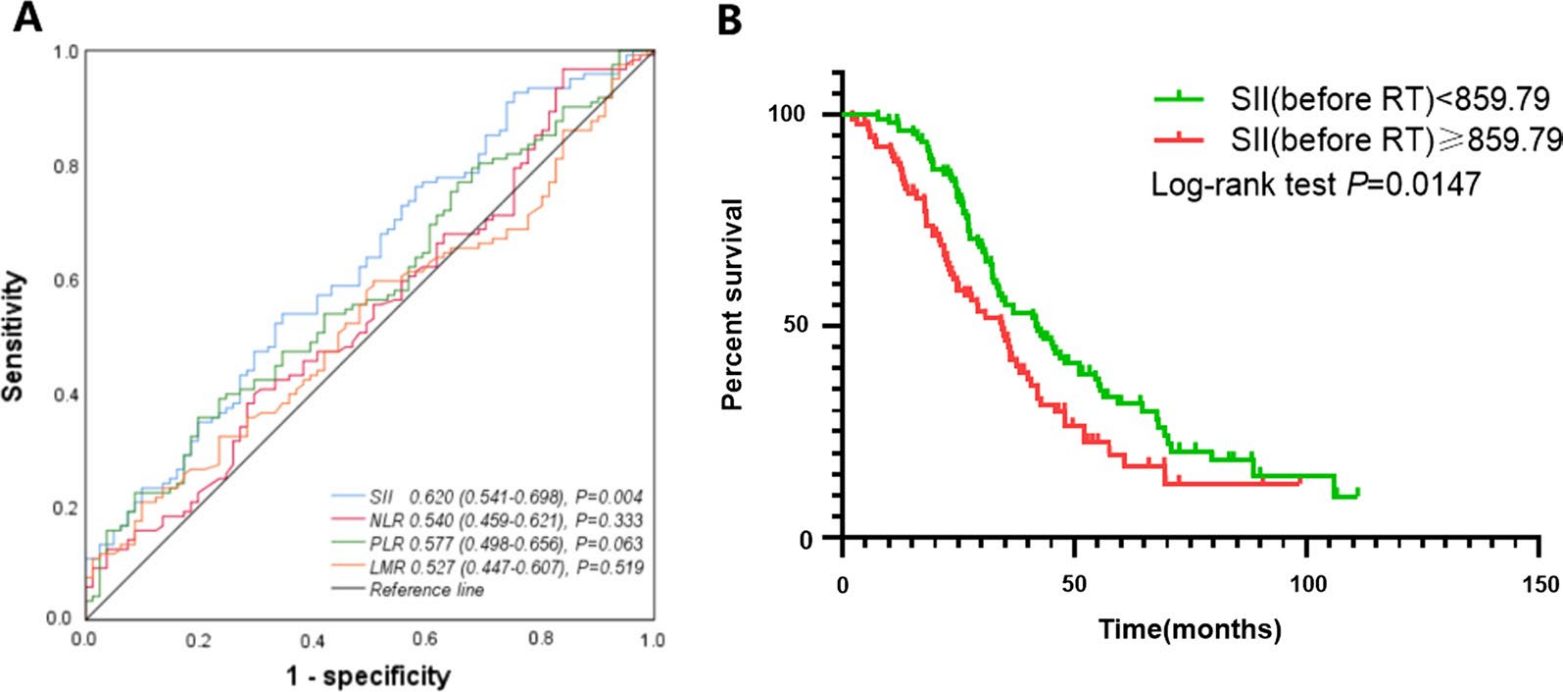
ROC was generated to evaluate the discriminatory ability of the SII, NLR, PLR and LMR (**A**); Kaplan–Meier curves of OS according to SII before brain radiotherapy (median overall survival, 42.1 vs. 34.5 months, HR (95%CI): 0.6653 (0.4708–0.9402), *P* < 0.05) (**B**)

The SII, with the area under the ROC curve of 0.620 (Fig. 1A, *P* = 0.004), was larger than NLR, PLR and LMR, which area were 0.540 (Fig. 1A, *P* = 0.333), 0.577 (Fig. 1A, *P* = 0.0.063) and 0.527 (Fig. 1A, *P* = 0.519), respectively. The optimal cutoff value of SII established by ROC curve analysis and the Youden Index was 859.79. Based on this cutoff, 93 (46%) patients had SII values of 859.79 or greater, who were assigned to the high SII group, and 109 (54%) patients with SII values less than 859.79 were included in the low SII group. According to the dynamic changes of SII within 1 week before the start of the radiation treatment and within 1 week before its completion, the numbers of patients in the high–high, high–low, low–low, and low–high groups were 59 (29.2%), 34 (16.8%), 67 (33.2%), and 42 (20.8%) participants, respectively. The correlations between the SII value and various clinicopathological features were not significant (Table 1).

### Survival outcomes for the entire study cohort

**Fig. 2:**
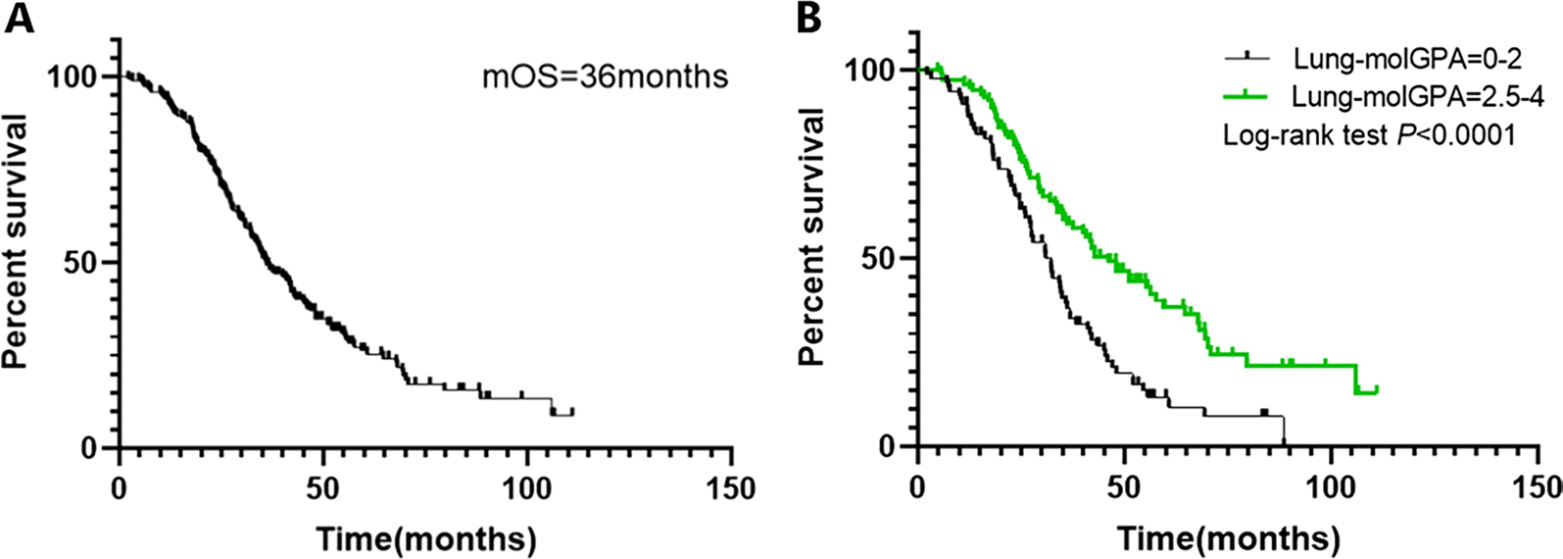
Overall survival (OS) of entire cohort (**A**) and OS of patients stratified according to Lung-molGPA (**B**)

At the end of the last follow-up, 139 (68.8%) patients had died, and the median OS of the entire cohort was 36 months (Fig. 2A). We used the Kaplan–Meier method to perform survival analyses and compared the survival differences between groups stratified by Lung-molGPA score, as this score has a significant impact on the prognosis of the patients. The median OS was significantly different between groups with Lung-molGPA scores 0–2 and 2.5–4 (32.2 months vs. 46.0 months, hazard ratio (HR): 1.935, 95% confidence interval (CI): 1.366–2.742, *P* < 0.05; Fig. 2B).

### Correlations of OS with SII value before brain radiotherapy and dynamic SII change for each subgroup

We first assessed the relationship between the SII value before brain radiotherapy and median OS, the results showed negative correlations between OS and SII in all cohorts, but the median survival of patients in the low and high groups were significantly different, with 42.1 and 34.5 months, respectively (HR: 0.6653, 95% CI: 0.4708–0.9402, *P* < 0.05; Fig. 1B).

**Fig. 3:**
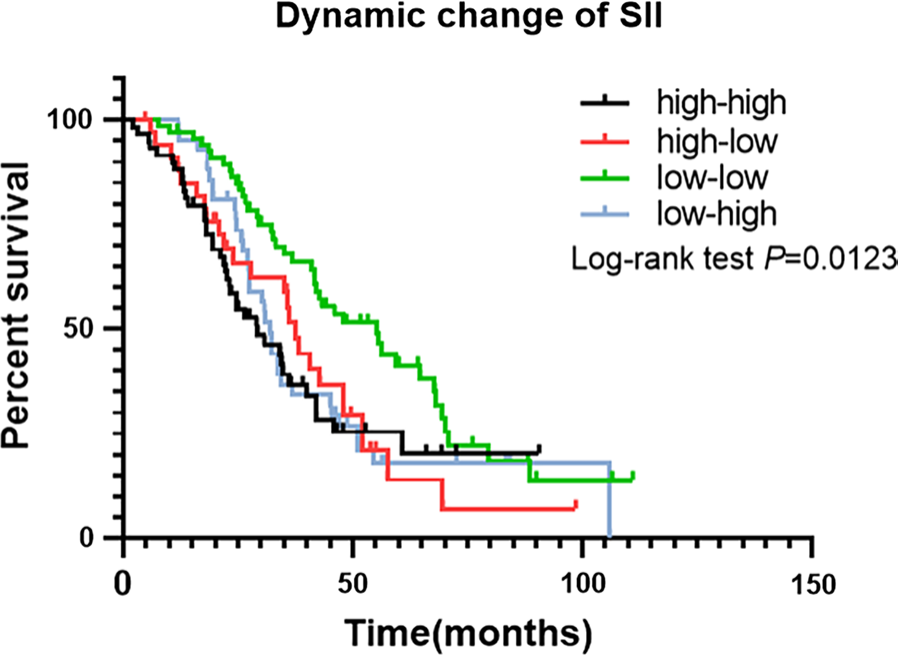
Kaplan–Meier analysis for OS of patients in four subgroups: high–high group, high–low group, low–high group, low–low group (median survival, 29.3 months vs. 37.5 months vs. 55.2 months vs. 32 months, *P* < 0.05)

**Fig. 4:**
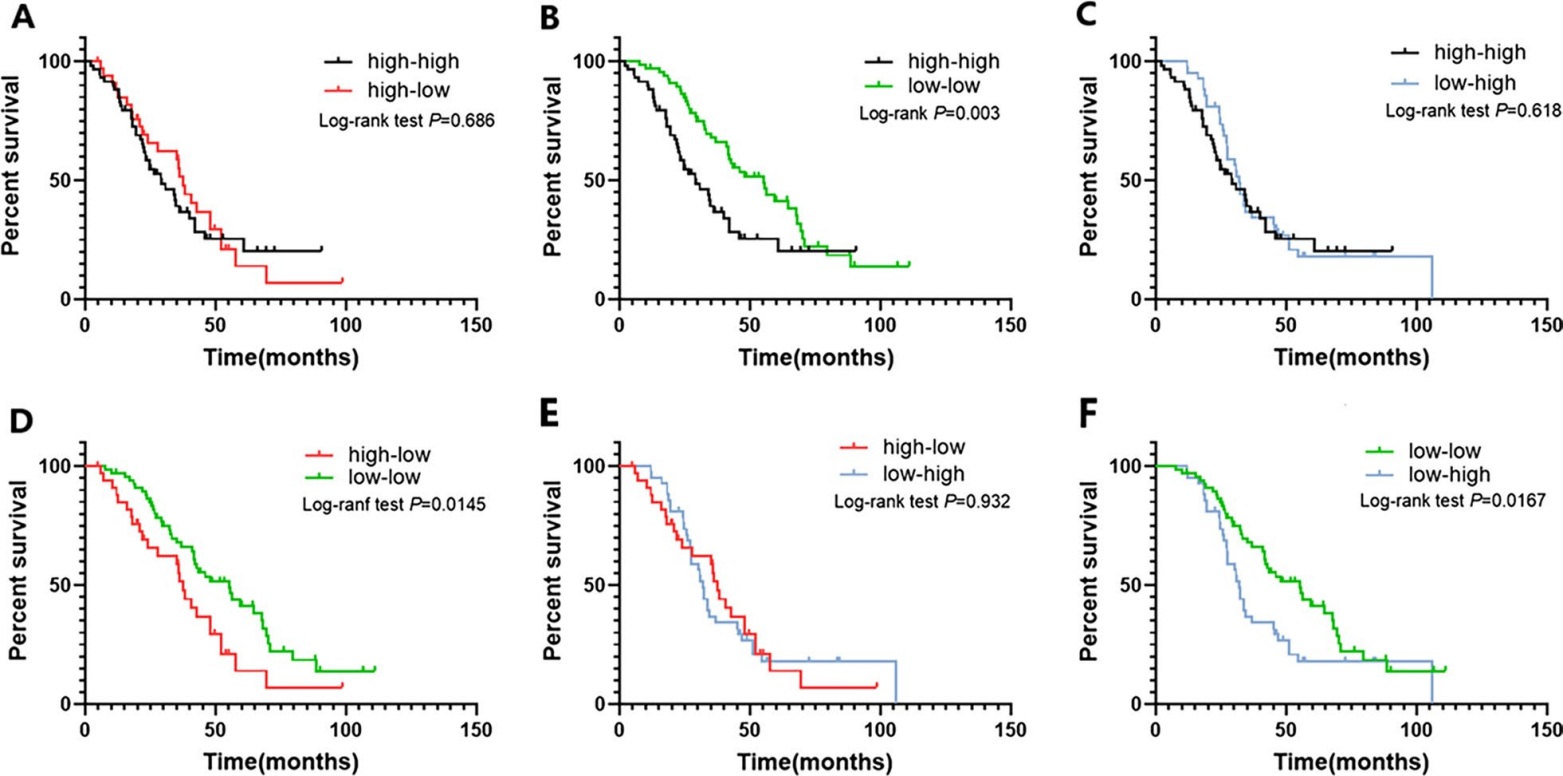
Kaplan–Meier analysis for OS of patients in four subgroups compared with each other (**A**–**F**)

Similarly, in the comparison of the four subgroups categorized according to the dynamic SII changes during BM radiotherapy, the OS rates of the patients in the different groups were significantly different (Fig. 3). The median survival time of patients in the low–low group was longest with 55.2 months, and in decreasing order, the median survival times of the high–low, low–high, and high–high groups were 37.5, 32.0, and 29.3 months, respectively (*P* < 0.05). The differences were statistically significant between the low–low and high–high groups (HR: 0.5285, 95% CI: 0.3322–0.8410, *P* = 0.003; Fig. 4B), the low–low and high–low groups (HR: 0.5470, 95% CI: 0.3134–0.9549, *P* = 0.014; Fig. 4D), and the low–low and low–high groups (HR: 0.5801, 95% CI: 0.3578–0.9405, *P* = 0.017; Fig. 4F). However, there was no significant difference between the remaining group pairings (Fig. 4A, C, E).

### Univariate and multivariate Cox regression analysis for OS

The results of the univariate and multivariate analyses regarding prognostic factors associated with OS are shown in Table 2. In univariate Cox regression analyses, the discriminating variables were sex (*P* = 0.862), age (*P* = 0.971), smoking status (*P* = 0.246), EGFR mutation (*P* = 0.371), Lung-molGPA (*P* < 0.001), brain radiation mode (*P* = 0.365), thoracic radiation (*P* = 0.010), chemotherapy (*P* = 0.114), and dynamic change of SII (*P* = 0.014). All variables with prognostic significance in the univariate analysis were further entered into the multivariate Cox regression analysis, and the results showed that dynamic change of SII (*P* = 0.032), Lung-molGPA (*P* < 0.001), and thoracic radiation (*P* = 0.048) were independent indicators of brain radiotherapy outcomes in patients with BM arising from EGFR-mutant lung adenocarcinoma.

**Table 2:** Univariate and multivariate Cox proportional hazard analysis of factors associated with OS

	Univariate analysis		Multivariate analysis	
	HR (95% CI)	*P* value	HR (95% CI)	*P* value
Sex	0.970 (0.687–1.369)	0.862	NA	NA
Age (years)	1.007 (0.702–1.443)	0.971	NA	NA
Smoking status	0.792 (0.535–1.174)	0.246	NA	NA
EGFR mutation	NA	0.371	NA	NA
Lung-molGPA	1.999 (1.423–2.808)	0.000	0.490 (0.345–0.696)	0.000
Brain radiation mode	NA	0.365	NA	NA
Thoracic radiation	1.588 (1.116–2.259)	0.010	0.698 (0.489–0.997)	0.048
Chemotherapy	1.429 (0.918–2.224)	0.114	NA	NA
Dynamic change of SII	NA	0.014	NA	0.032

## Discussion

Lung adenocarcinoma patients with EGFR mutations have a higher incidence of BM, both at initial diagnosis and during the course of the disease, usually leading to poor quality of life and survival prognosis, even after treatment with TKIs [16]. The Lung-molGPA can predict the prognosis of patients with BM from NSCLC and facilitate clinical decision-making [17]. In our study, higher Lung-molGPA scores corresponded to a better prognosis, which is consistent with the results of previous studies. However, Lung-molGPA is a prognostic factor that may have little value for predicting the efficacy of brain radiotherapy. Brain radiotherapy plays an important role in the management of EGFR-mutant NSCLC patients with BM [18, 19]. A reliable factor to predict the survival and prognosis of these patients after cranial radiotherapy is still lacking.

Inflammation is a recognized hallmark of cancer, and inflammatory cells substantially contribute to the development, spread, and metastasis of malignancies [20, 21]. In past decades, inflammation has been shown to play a critical role in tumorigenesis after the first indication of a possible link between inflammation and cancer had been provided in the nineteenth century. Current research indicates that radiation can recruit inflammatory cells into the tumor microenvironment by stimulating the release of signals and chemokines, thereby invoking immune responses to support the survival of cancer cells or promote their death [22–24].

SII is a novel and integrated systematic inflammation index based on neutrophil, platelet, and lymphocyte counts. In the peripheral blood of patients who develop different types of cancers, the numbers of neutrophils and platelets increase remarkably, and these cells produce inflammatory cytokines and chemokines, which may contribute directly to malignant progression [25–27]. Although lymphocytes in tumors may not always be active due to immune escape or tolerance, increased infiltration is associated with a better prognosis in cancer patients [28]. Therefore, it is concluded that inflammation plays a crucial role in the development and progression of tumors, and SII may be a reliable prognostic index for the survival of cancer patients. Compared with other existing indexes based on inflammation, such as NLR, PLR, and LMR, SII was a more comprehensive and reliable indicator which had more value in predicting OS than other indices in this study. The prognostic role of SII has been demonstrated in patients with NSCLC, including NSCLC patients with BM harboring EGFR mutations and patients receiving treatment with EGFR-TKIs, and elevated SII values indicated a worse OS [29, 30]. Because of the tight correlation between radiotherapy and immune status, we hypothesized that SII may be related to the efficacy of radiotherapy.

In this study, we explored the prognostic role of SII in EGFR-mutant lung adenocarcinoma patients who received BM radiotherapy, and the results were consistent with those described above. We chose the pre-radiotherapy SII of patients to obtain the optimal SII cutoff value and found that patients with SII > 859.79 had a worse prognosis than patients with lower SII values. Although the cutoff value of SII is not consistent across various studies, the results all show that the SII value is significantly negatively associated with OS. The reasons we chose the pre-radiotherapy SII to determine the optimal cutoff value is to rule out any radiotherapy effect on the inflammatory state of the body and to observe the effects of changes in SII before and after radiotherapy on patient prognosis.

Tumor cells are constantly evolving to establish a network of cellular and soluble components, which can induce several inhibitory mechanisms, thus allowing immune evasion and promoting tumor progression [31, 32]. The malignant tumor, like any dynamic system, undergoes adaptive changes in response to external influences, including treatment, especially during targeted therapy or chemotherapy and radiotherapy, which may be related to tumor tolerance to these interventions. For instance, most patients with EGFR mutations will eventually develop a progressive disease course within about 1 year of EGFR-TKI treatment because of the most common mechanism—development of acquired EGFR T790M mutation, and approximately 3–10% of acquired resistance to EGFR-TKIs is associated with histologic transformation to small cell lung cancer [33, 34]. Radiotherapy, as a major method of treatment for cancer patients, can trigger immune-mediated tumor responses and remodel the inflammatory microenvironment [35, 36]. Therefore, we speculated that the SII may also change during radiotherapy, and different dynamic change patterns may be closely related to the prognosis of patients.

We extended the application of the SII by analyzing changes in its value. The result showed that the prognosis of patients was significantly better in the low–low group than in all other groups, which indicated that the pathophysiology associated with a persistently low SII provided an environment more unfavorable for tumor growth, infiltration, and metastasis. At the same time, it reminds us that patients whose SII is persistently high need to be considered for further maintenance treatment and should be followed closely, which can help to detect recurrence and metastasis earlier in these patients and improve their prognosis. Excluding the low–low group, intergroup comparisons of the other three groups were not significant, possibly because the total number of patients in this study was relatively small and the number of people assigned to each group was not equal. However, in univariate and multivariate Cox regression analyses, dynamic SII changes proved to be an independent prognostic factor of OS.

Our research has some limitations. First, it was more susceptible to potential biases because this is a retrospective study. Second, the number of patients enrolled in this study was relatively small, so the sample size in subgroup analyses might not be sufficient to be representative. Third, different systemic regimens among subgroups may have affected the survival analysis. Hence, multicenter prospective studies with larger sample sizes are needed to verify the prognostic value of SII in this field.

## Conclusions

As a biomarker reflecting the systemic immune-inflammation status, the SII and its dynamic change may have a prognostic value in patients with EGFR-mutant lung adenocarcinoma treated with BM radiotherapy, which seemed to be a promising parameter for inclusion in future prognostic systems.

## Data Availability

The datasets generated for this study are available on request to the corresponding author.

## Abbreviations

BM: Brain metastases
CI: Confidence interval
EGFR: Epidermal growth factor receptor
HR: Hazard ratio
NSCLC: Non-small cell lung cancer
OS: Overall survival
ROC: Receiver operating characteristic
SII: Systemic immune-inflammation index
TKI: Tyrosine kinase inhibitor

## References

[1] Soria J-C, Ohe Y, Vansteenkiste J, Reungwetwattana T, Chewaskulyong B, Lee KH (2018). Osimertinib in untreated EGFR-mutated advanced non-small-cell lung cancer. N Engl J Med.

[2] Rangachari D, Yamaguchi N, VanderLaan PA, Folch E, Mahadevan A, Floyd SR (2015). Brain metastases in patients with EGFR-mutated or ALK-rearranged non-small-cell lung cancers. Lung Cancer.

[3] Han G, Bi J, Tan W, Wei X, Wang X, Ying X (2016). A retrospective analysis in patients with EGFR-mutant lung adenocarcinoma: is EGFR mutation associated with a higher incidence of brain metastasis?. Oncotarget.

[4] Khalifa J, Amini A, Popat S, Gaspar LE, Faivre-Finn C (2016). Brain metastases from NSCLC: radiation therapy in the era of targeted therapies. J Thorac Oncol.

[5] Chen Y, Wei J, Cai J, Liu A (2019). Combination therapy of brain radiotherapy and EGFR-TKIs is more effective than TKIs alone for EGFR-mutant lung adenocarcinoma patients with asymptomatic brain metastasis. BMC Cancer.

[6] Greten FR, Grivennikov SI (2019). Inflammation and cancer: triggers, mechanisms, and consequences. Immunity.

[7] Van Berckelaer C, Van Geyt M, Linders S, Rypens C, Trinh XB, Tjalma WAA (2020). A high neutrophil–lymphocyte ratio and platelet–lymphocyte ratio are associated with a worse outcome in inflammatory breast cancer. Breast.

[8] Peng H, Luo X (2019). Prognostic significance of elevated pretreatment systemic inflammatory markers for patients with prostate cancer: a meta-analysis. Cancer Cell Int.

[9] Li B, Wang S, Li C, Guo M, Xu Y, Sun X (2019). The kinetic changes of systemic inflammatory factors during bevacizumab treatment and its prognostic role in advanced non-small cell lung cancer patients. J Cancer.

[10] Yang R, Chang Q, Meng X, Gao N, Wang W (2018). Prognostic value of systemic immune-inflammation index in cancer: a meta-analysis. J Cancer.

[11] Chen J-H, Zhai E-T, Yuan Y-J, Wu K-M, Xu J-B, Peng J-J (2017). Systemic immune-inflammation index for predicting prognosis of colorectal cancer. World J Gastroenterol.

[12] Hu B, Yang X-R, Xu Y, Sun Y-F, Sun C, Guo W (2014). Systemic immune-inflammation index predicts prognosis of patients after curative resection for hepatocellular carcinoma. Clin Cancer Res.

[13] Murthy P, Zenati MS, Al Abbas AI, Rieser CJ, Bahary N, Lotze MT (2020). Prognostic value of the systemic immune-inflammation index (SII) after neoadjuvant therapy for patients with resected pancreatic cancer. Ann Surg Oncol.

[14] Gao Y, Zhang H, Li Y, Wang D, Ma Y, Chen Q (2018). Preoperative increased systemic immune-inflammation index predicts poor prognosis in patients with operable non-small cell lung cancer. Clin Chim Acta.

[15] Deng G, Zhang Y, Ke J, Wang Q, Qin H, Li J (2021). Effect of brain radiotherapy strategies on prognosis of patients with EGFR-mutant lung adenocarcinoma with brain metastasis. J Transl Med.

[16] Rosell R, Moran T, Queralt C, Porta R, Cardenal F, Camps C (2009). Screening for epidermal growth factor receptor mutations in lung cancer. N Engl J Med.

[17] Sperduto PW, Yang TJ, Beal K, Pan H, Brown PD, Bangdiwala A (2017). Estimating survival in patients with lung cancer and brain metastases: an update of the graded prognostic assessment for lung cancer using molecular markers (Lung-molGPA). JAMA Oncol.

[18] Rosell R, Karachaliou N (2017). Brain metastases in patients with EGFR-mutant non-small-cell lung cancer. Lancet Respir Med.

[19] Magnuson WJ, Lester-Coll NH, Wu AJ, Yang TJ, Lockney NA, Gerber NK (2017). Management of brain metastases in tyrosine kinase inhibitor-naïve epidermal growth factor receptor-mutant non-small-cell lung cancer: a retrospective multi-institutional analysis. J Clin Oncol.

[20] Diakos CI, Charles KA, McMillan DC, Clarke SJ (2014). Cancer-related inflammation and treatment effectiveness. Lancet Oncol.

[21] Coussens LM, Werb Z (2002). Inflammation and cancer. Nature.

[22] Weichselbaum RR, Liang H, Deng L, Fu Y-X (2017). Radiotherapy and immunotherapy: a beneficial liaison?. Nat Rev Clin Oncol.

[23] Brooks ED, Chang JY (2019). Time to abandon single-site irradiation for inducing abscopal effects. Nat Rev Clin Oncol.

[24] Demaria S, Golden EB, Formenti SC (2015). Role of local radiation therapy in cancer immunotherapy. JAMA Oncol.

[25] Singh N, Baby D, Rajguru J, Patil P, Thakkannavar S, Pujari V (2019). Inflammation and cancer. Ann Afr Med.

[26] Mollinedo F (2019). Neutrophil degranulation, plasticity, and cancer metastasis. Trends Immunol.

[27] Schlesinger M (2018). Role of platelets and platelet receptors in cancer metastasis. J Hematol Oncol.

[28] Ozkan EE, Kaymak Cerkesli ZA, Erdogan M (2020). Predictive value of immune-inflammation indices in metabolic response and outcome after curative radiotherapy in patients with non-small cell lung cancer. Clin Respir J.

[29] Li H, Wang G, Zhang H, Song X, Cao J, Zhang X (2019). Prognostic role of the systemic immune-inflammation index in brain metastases from lung adenocarcinoma with different EGFR mutations. Genes Immun.

[30] Ying H-Q, Liao Y-C, Luo Y-R, Xiong G, Huang Y, Nie R-W (2021). Cancer-elicited inflammation attenuates response and outcome in tyrosine kinase inhibitor naive patients with advanced NSCLC. Pharmacol Res.

[31] Teng MWL, Galon J, Fridman W-H, Smyth MJ (2015). From mice to humans: developments in cancer immunoediting. J Clin Invest.

[32] Saleh R, Elkord E (2020). Acquired resistance to cancer immunotherapy: role of tumor-mediated immunosuppression. Semin Cancer Biol.

[33] Wu S-G, Shih J-Y (2018). Management of acquired resistance to EGFR TKI-targeted therapy in advanced non-small cell lung cancer. Mol Cancer.

[34] Marcoux N, Gettinger SN, O’Kane G, Arbour KC, Neal JW, Husain H (2019). EGFR-mutant adenocarcinomas that transform to small-cell lung cancer and other neuroendocrine carcinomas: clinical outcomes. J Clin Oncol.

[35] Herrera FG, Bourhis J, Coukos G (2017). Radiotherapy combination opportunities leveraging immunity for the next oncology practice: radiation–immunotherapy combinations. CA Cancer J Clin.

[36] McLaughlin M, Patin EC, Pedersen M, Wilkins A, Dillon MT, Melcher AA (2020). Inflammatory microenvironment remodelling by tumour cells after radiotherapy. Nat Rev Cancer.

